# Integrative bioinformatics analysis of biomarkers and pathways for exploring the mechanisms and molecular targets associated with pyroptosis in type 2 diabetes mellitus

**DOI:** 10.3389/fendo.2023.1207142

**Published:** 2023-11-15

**Authors:** Wei Wang, Yao Wang

**Affiliations:** ^1^ Department of Endocrinology, School of Medicine, Zhongda Hospital, Institute of Diabetes, Southeast University, Nanjing, Jiangsu, China; ^2^ Department of Endocrinology, First Affiliated Hospital of Baotou Medical Collage, Baotou, China

**Keywords:** diabetes mellitus, pyroptosis, immune infiltration, risk prediction, mechanisms, biomarkers

## Abstract

**Introduction:**

Research has shown that pyroptosis contributes greatly to the progression of diabetes and its complications. However, the exact relationship between this particular cell death process and the pathology of type 2 diabetes mellitus (T2DM) remains unclear. In this study, we used bioinformatic tools to identify the pyroptosis-related genes (PRGs) associated with T2DM and to analyze their roles in the disease pathology.

**Methods:**

Two microarray datasets, GSE7014 and GSE25724, were obtained from the GEO database and assessed for differentially expressed genes (DEGs). The T2DM-associated DEGs that overlapped with differentially expressed PRGs were noted as T2DM-PRGs. Subsequently, 25 T2DM-PRGs were validated and subjected to functional enrichment analysis through Gene Ontology annotation analysis, Kyoto Encyclopedia of Genes and Genomes pathway analysis, and gene set enrichment analysis (GSEA). The diagnostic and predictive value of the T2DM-PRGs was evaluated using receiver operating characteristic curves (ROC). Additionally, a single-sample GSEA algorithm was applied to study immune infiltration in T2DM and assess immune infiltration levels.

**Results:**

We identified 25 T2DM-PRGs that were significantly enriched in the nuclear factor-kappa B signaling and prostate cancer pathways. The top five differentially expressed prognostic T2DM-PRGs targeted by miRNAs were *PTEN*, *BRD4*, *HSP90AB1*, *VIM*, and *PKN2*. The top five differentially expressed T2DM-PRGs associated with transcription factors were *HSP90AB1*, *VIM*, *PLCG1*, *SCAF11*, and *PTEN*. The genes *PLCG1*, *PTEN*, *TP63*, *CHI3L1*, *SDHB*, *DPP8*, *BCL2*, *SERPINB1*, *ACE2*, *DRD2*, *DDX58*, and *BTK* showed excellent diagnostic performance. The immune infiltration analysis revealed notable differences in immune cells between T2DM and normal tissues in both datasets. These findings suggest that T2DM-PRGs play a crucial role in the development and progression of T2DM and could be used as potential diagnostic biomarkers and therapeutic targets.

**Discussion:**

Investigating the mechanisms and biomarkers associated with pyroptosis may offer valuable insights into the pathophysiology of T2DM and lead to novel therapeutic approaches to treat the disease.

## 1 Introduction

Approximately 285 million people worldwide have either type 1 or type 2 diabetes mellitus (DM), which constitutes a severe global health problem owing to the high morbidity and increasing clinical impact caused by these diseases. The notable health and economic consequences of DM warrant the search for effective treatment options (1). Specifically, type 2 diabetes mellitus (T2DM) is a complex metabolic disorder that is characterized by hyperglycemia caused by insulin resistance and deficiency (2). However, the biomolecules and signaling pathways involved in the pathogenesis of the disease remain poorly understood. Owing to the multiple processes and variables contributing to DM, it is imperative that key biomolecules are discovered to act as prospective therapeutic targets and enhance treatment strategies.

Pyroptosis is a newly discovered type of cell death process that is associated with inflammatory reactions. Its mechanism of action differs from that of apoptosis and necrosis in several ways. Some notable features of pyroptosis include the proteolytic activity of caspase-1, -4, -5, or -11 as well as the secretion of proinflammatory cytokines, such as interleukin (IL)-1 and IL-18, which can lead to the accumulation of immune cells (3, 4). During pyroptosis, many pores develop on the cell membrane, resulting in membrane destabilization and the subsequent swelling and lysis of the cell due to the entry of ions and water (5). Therefore, pyroptosis poses a dilemma for the innate immune system. Additionally, although pyroptosis prevents bacterial infiltration in multicellular organisms, excessive activation of this cell death process may result in chronic inflammation (6). This apparent contradiction can be explained by the differences in the aggressive strategies utilized and the specific cells affected by different pathogens (3). Further studies on pyroptosis may aid the discovery of novel therapies for immunological disorders and other diseases.

Research has shown that pyroptosis contributes greatly to the progression of DM and its complications (7–9). One study revealed that hyperglycemia could increase the inflammatory response and cellular pyroptosis in a mouse model of diabetes, thereby inducing significant muscle cell loss and tissue remodeling (10). However, the exact relationship between pyroptosis and the pathology of T2DM remains to be fully elucidated.

Bioinformatics is an important computational tool for evaluating gene expression data and identifying the target genes and molecular mechanisms involved in various illnesses. With the advancement and increasing utilization of high-throughput technologies in the biomedical research fields, integrative bioinformatics has become a promising approach for investigating the mechanisms underlying T2DM and biomolecular targets for its treatment.

Previous studies have used bioinformatics to identify potential genes involved in the pathogenesis of T2DM (11–13). In this study, we investigated the relationship between pyroptosis-related genes (PRGs) and T2DM using various computational tools. We identified 25 PRGs associated with T2DM (hereinafter T2DM-PRGs) from the Gene Expression Omnibus (GEO) and PubMed databases and conducted functional enrichment analysis of those genes to further understand their roles in T2DM. The diagnostic and predictive value of the T2DM-PRGs was evaluated using receiver operating characteristic (ROC) curves, and their association with immune infiltration in T2DM was analyzed. Our exploration of pyroptosis in T2DM increases our understanding of the pathogenesis of the disease and provides a new approach to its treatment.

## 2 Materials and methods

### 2.1 Data preprocessing and differentially expressed genes

**Figure 1:**
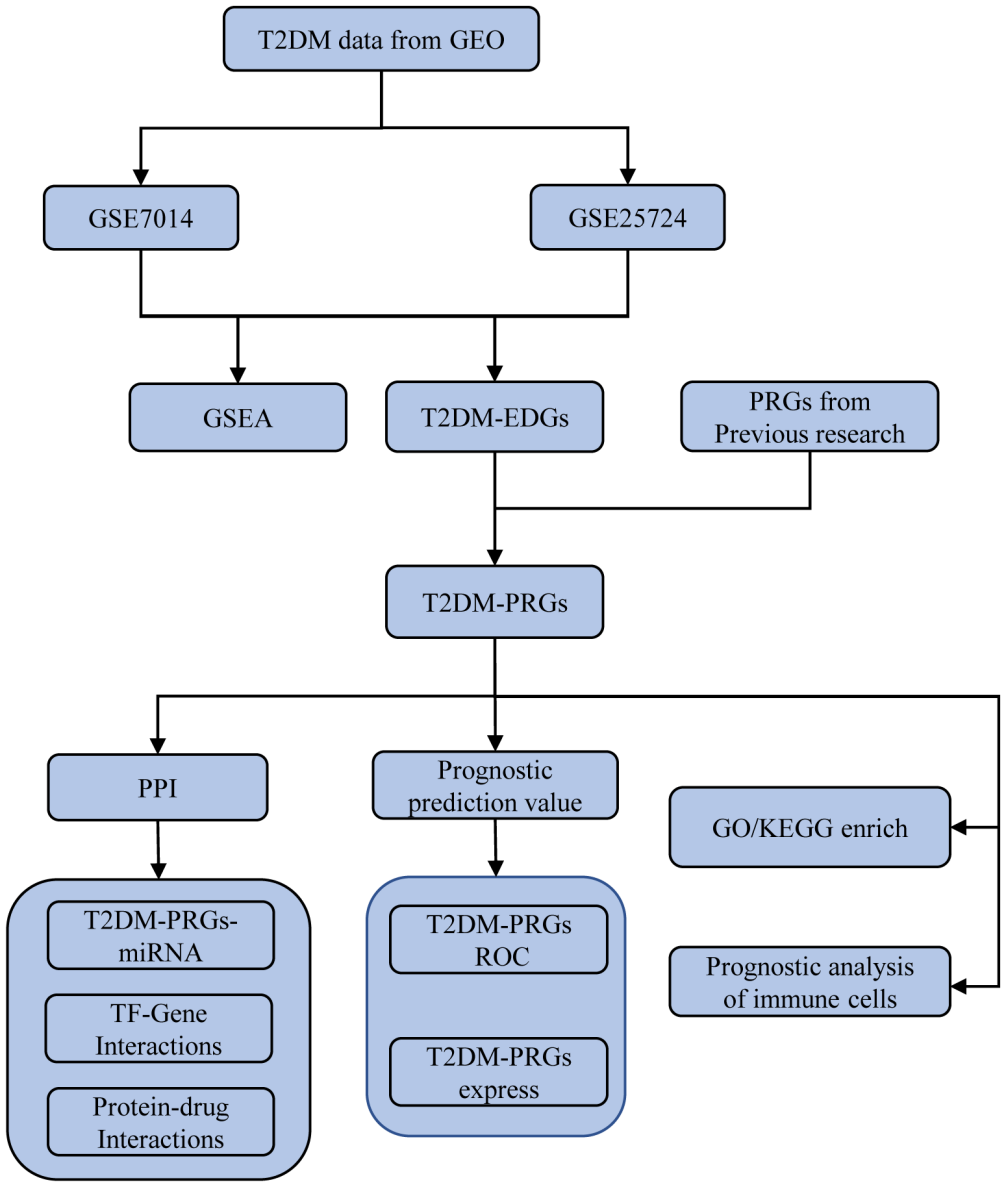
Diagram of the study workflow.

The workflow for this study is presented in **Figure 1**. The GEOquery package in R (14) was used to extract the gene expression profile datasets GSE7014 (15) and GSE25724 (16) for T2DM from the GEO database (17). The 20 T2DM and six normal (non-diabetic) tissue samples of the GSE7014 dataset were from human (*Homo sapiens*) skeletal muscle biopsies, and their genomes had been sequenced using the GPL570 [HG-U133_Plus_2] Affymetrix Human Genome U133 Plus 2.0 Array platform. The six T2DM and seven normal samples of the GSE25724 dataset were human (*Homo sapiens*) pancreatic islet tissues, and the genes had been sequenced using the GPL96 [HG-U133A] Affymetrix Human Genome U133A Array platform. Both datasets had a common sequencing type, sample grouping information, and species source, with sufficient sample size and data quality. This was crucial for our analysis.

The limma package in R (18) was used to perform a differential analysis of the groupings on the basis of the gene expression levels in the T2DM and normal tissues. This analysis allowed for the identification of differentially expressed genes (DEGs) and their effect on the development of T2DM. First, the samples were normalized. We applied a set of filtering criteria to determine the differentially expressed PRGs. Specifically, we defined upregulated genes as those with a log fold change (FC) value of greater than 0.5 and an adjusted p-value of less than 0.05. Conversely, genes with a logFC of less than 0.5 and an adjusted p-value of less than 0.05 were classified as downregulated genes.

Additionally, we conducted a comprehensive search for pyroptosis-related studies on the PubMed database (19–33). The retrieved data were then combined with information obtained from the GeneCards database (34), AmiGO2 database (35), and Molecular Signatures Database (MSigDB) (36). This helped us identify a total of 356 PRGs (**Supplementary Table 1**).

Next, the genes related to T2DM were obtained by intersecting the DEGs between the disease and normal groups in the two datasets. Likewise, the T2DM-PRGs were identified by examining the intersection of DEGs associated with both T2DM and PRGs.

### 2.2 Functional enrichment analysis

Functional and pathway enrichment analyses of the T2DM-PRGs were conducted using Gene Ontology (GO) (37) and Kyoto Encyclopedia of Genes and Genomes (KEGG) (38) database tools, respectively. GO analysis is a common method for studying the function of genes, whereas KEGG provides information on biological pathways, diseases, and drugs. The clusterProfiler package in R (39) was used to analyze the data, with the significance threshold set at a p-value of less than 0.05.

### 2.3 Gene set enrichment analysis

The gene set enrichment analysis (GSEA) method was used to assess the distribution pattern of genes within a predetermined set in the gene table. This was done by ranking the genes according to their correlation with the genotype in order to determine their impact on the phenotype (40). We acquired the gene sets “c2.kegg.v7.2.symbols” and “c5.go.v7.2.symbols” from MSigDB (39) and performed GSEA on them using the clusterProfiler package in R (39), where a p-value of less than 0.05 was considered statistically significant.

### 2.4 Construction of a protein–protein interaction network

The STRING database (41) is used to search for known proteins and protein–protein interactions (PPIs) and includes 2031 species, 1.38 million PPIs, and 9.6 million proteins. It contains outcomes derived from experimental data, results compiled through text mining of PubMed abstracts and other databases, and results forecasted using bioinformatic techniques. Using the STRING database, we constructed a PPI network for the DEGs associated with T2DM-PRGs.

### 2.5 Construction of interaction networks between T2DM-PRGs and related miRNAs, transcription factors, and drugs

The NetworkAnalyst database (42) is a platform used for visualizing gene expression profiles and meta-analytic data. It supports various data types from 17 species, including single or multiple gene or protein lists, single RNA sequencing or microarray gene expression data tables, multiple gene expression tables, network files, and other upload formats. In this study, we focused on analyzing the control of gene expression by miRNAs and transcription factors (TFs) at the post-transcriptional stage to identify diseases associated with the target genes (43, 44). The TarBase v8.0 (45) and ENCODE databases (46) were used to identify miRNAs and TFs associated with the differentially expressed T2DM-PRGs. We used the DrugBank v5.0 database (47) to predict the correlation between the target genes and drugs. The target DEG–miRNA, DEG–TF, and DEG–drug networks of T2DM-PRGs were visualized using Cytoscape software (48).

### 2.6 Pyroptosis-related gene expression analysis and ROC validation

We analyzed the expression levels of T2DM-PRGs in both the disease and normal groups, using box plots to visualize the results. ROC curves were used to evaluate the diagnostic and predictive value of those genes, where an area under the ROC curve (AUC) value of greater than 0.7 was deemed accurate for predictive purpose. The AUC cutoff value of 0.7 is a commonly used threshold for assessing the diagnostic accuracy of a test or model. The AUC takes values from 0.5 to 1, where 0.5 indicates no discriminatory ability and 1 indicates full discriminatory ability. An AUC value of greater than 0.7 implies reasonable accuracy in diagnosing a situation.

### 2.7 Analysis of the association between immune infiltration and pyroptosis-related genes

Most tumor microenvironments consist of a combination of immune and inflammatory cells, tumor-associated fibroblasts, interstitial tissue, and various cytokines and chemokines that surround the tumor tissue. An essential part of disease research and therapy prognosis prediction is the examination of immune cell infiltration in the affected tissues.

As an extension of the GSEA method, single-sample GSEA provides the degree of enrichment of the gene set in each sample from the input data by defining the enrichment score (49). In this study, the single-sample GSEA method was used to compare immune infiltration between the diseased and normal tissues, to examine the relative increase or decrease in the occurrence of two diseases compared with the general population, and to assess the relationship between T2DM-PRGs and immune cells. Pearson’s correlation analysis was used to identify the association between the T2DM-PRGs and the level of immune invasion.

### 2.8 Statistical analysis

We used R v4.1.2 software for all data processing and statistical analyses. An independent Student’s *t*-test was applied to normally distributed variables to compare two continuous variables, whereas the Mann–Whitney U test (Wilcoxon rank sum) was used to assess differences between non-normally distributed variables. The pROC package in R was used to plot the ROC curve, and the AUC was calculated to predict patient prognosis. Statistical significance was defined as a p-value of less than 0.05.

Owing to the large number of statistical comparisons performed, multiple hypothesis testing corrections were carried out to control the probability of false discovery. Common multiple testing correction methods include the Bonferroni and Benjamini-Hochberg (also known as false discovery rate correction) procedures, which correct the original significance threshold on the basis of the sample size and desired significance level to control the overall probability of false discovery. In this study, the Bonferroni method was used for multiple testing corrections. Cross-validation and repeated experiments were used to assess the consistency and stability of the statistical model across different datasets. These help to determine whether the results are highly reliable and reduce the likelihood of false positives. Setting the appropriate significance level (e.g., 0.05 or 0.01) is an important factor in controlling the false discovery rate. Tighter significance levels will reduce the likelihood of false discoveries and lead to the omission of true differences. Independent validation was performed for the important DEGs to verify their expression or functional changes in different sample sets using other experimental methods. Additionally, biological functional analysis was conducted to further validate the biological significance of the differences, to exclude false-positive results.

## 3 Results

### 3.1 Differential expression analysis

**Figure 2:**
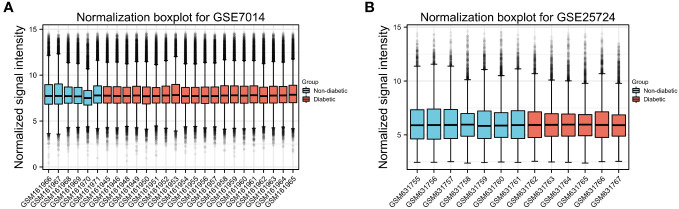
Normalized box plots of the GSE7014 and GSE25724 dataset samples. **(A, B)** Blue represents the normal group, and pink represents the disease group. The abscissa in the figure represents the sample number, and the ordinate represents the chip signal intensity. The signal intensity of each sample in the two datasets was approximately at the median level, indicating a good degree of sample normalization.

**Figure 3:**
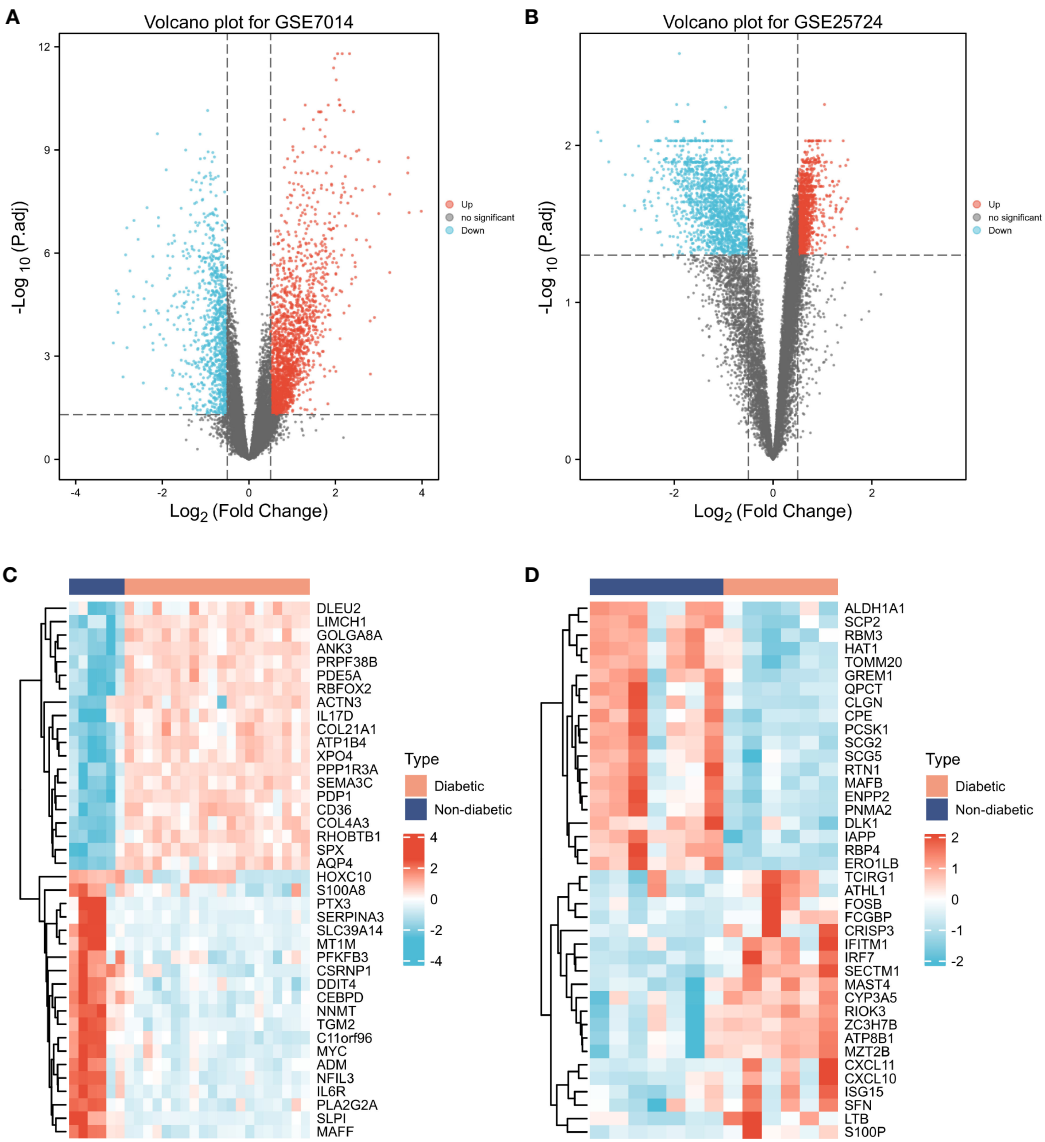
Differentially expressed genes (DEGs). **(A, B)** Volcano plot of T2DM-related DEGs in the GSE7014 and GSE25724 datasets. The abscissa is the log2 fold change, the ordinate is –log10 (adjusted p-value), red nodes represent upregulated DEGs, blue nodes represent downregulated DEGs, and grey nodes represent genes that are not significantly differentially expressed. **(C, D)** Heatmaps of T2DM-related DEGs in the GSE7014 and GSE25724 datasets. The horizontal axis indicates the patient ID, the vertical axis indicates the respective DEGs, red represents high gene expression, blue represents low gene expression, pink bars indicate normal tissue, and blue bars indicate T2DM tissue.

**Figure 4:**
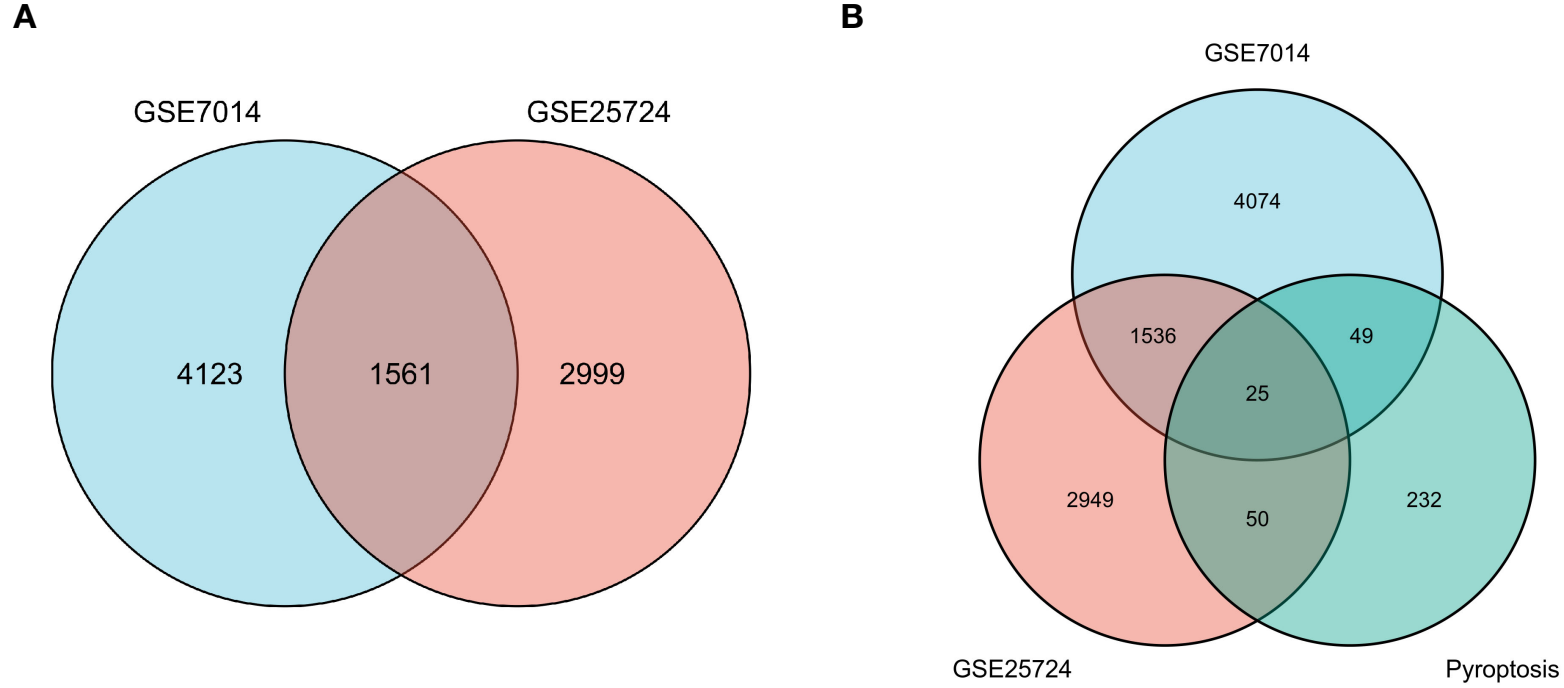
Venn diagrams of differentially expressed genes (DEGs). **(A)** The blue circle represents the DEGs of GSE7014, and the pink circle represents the DEGs of GSE25724. The Venn diagram was constructed with a log fold change > 0.5 and p < 0.05 as the thresholds, and 1561 T2DM-related genes were obtained in the overlapping region. **(B)** The blue circle indicates DEGs in GSE7014, the pink circle indicates DEGs in GSE25724, and the pistachio circle indicates genes related to pyroptosis. Twenty-five T2DM-associated DEGs in common with pyroptosis-related genes were obtained: *SCAF11*, *PKN2*, *ELAVL1*, *BRD4*, *UBR2*, *PRF1*, *PLCG1*, *PTEN*, *TP63*, *CHI3L1*, *SDHB*, *DPP8*, *BCL2*, *TRIM31*, *METTL3*, *SERPINB1*, *ACE2*, *FOXO1*, *DRD2*, *DDX58*, *VIM*, *BTK*, *HSP90AB1*, *NLRP1*, and *PRKACA*.

First, normalization of the sample data in the two datasets was performed, and box plots of the normalized data were constructed (**Figures 2A, B**). To examine the impact of gene expression levels on T2DM tissues relative to normal tissues, the differential analysis package limma was used to generate DEGs for both datasets, which were then visualized in volcano plots (**Figures 3A, B**). In the GSE7014 dataset, 5684 DEGs were identified, 1807 of which were upregulated and 3877 were downregulated. A classification heatmap was generated (**Figure 3C**), where the DEGs were classified into two groups: diabetic and non-diabetic. In the GSE25724 dataset, 4560 DEGs were identified, 2373 of which were upregulated and 2187 were downregulated. The heatmap of the classified DEGs is shown in **Figure 3D**. By comparing the DEGs from both datasets, we identified 1561 genes that are associated with T2DM (**Figure 4A**). Moreover, by intersecting the PRGs with the DEGs from both datasets, we identified 25 T2DM-associated DEGs and PRGs in common (**Figure 4B**; **Supplementary Table 1**).

### 3.2 Functional enrichment analysis of the differentially expressed T2DM-PRGs

**Figure 5:**
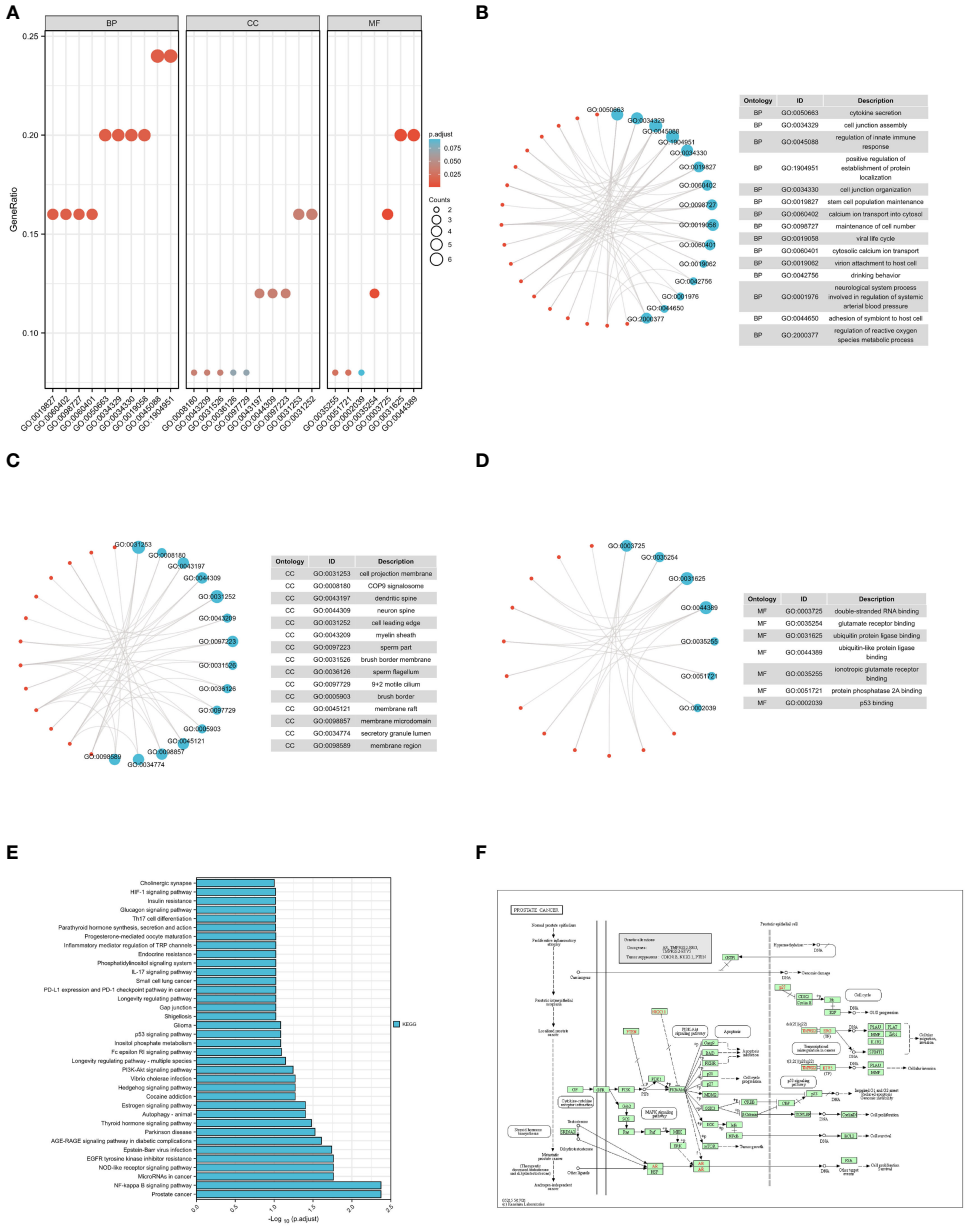
GO and KEGG enrichment analyses. **(A)** GO enrichment analysis divides gene functions into three categories: biological processes (BP), cellular components (CC), and molecular functions (MF). The ordinate indicates the –log(adjusted p-value), the abscissa represents GO terms (enriched items), and the bar colors indicate the activation (red) or inhibition (blue) of GO terms. **(B, C)** The first 15 items of the BP and CC categories are displayed. Blue nodes represent the items, red nodes represent the molecules, and lines represent the relationships between the items and molecules. Lines indicate that the corresponding molecules have annotations for the corresponding items. The node size corresponds to the total number of intersections of molecules within the ID entry for the molecules entered. **(D)** The seven items of the MF category are displayed; blue nodes represent the items, red nodes represent the molecules, and lines represent the relationship between the items and molecules. Lines indicate that the corresponding molecule has an annotation for the corresponding item. The node size corresponds to the total number of intersections of molecules within the ID entry for the molecules entered. **(E)** KEGG pathway enrichment analysis. The abscissa indicates the gene ratio, the ordinate indicates the pathway name, and the height of the column represents the size of the –log10(p-value); the higher the ID, the higher the reliability. **(F)** Significantly enriched KEGG pathways.

To examine the connections between the differentially expressed T2DM-PRGs and various biological processes, molecular functions, cellular components, biological pathways, and diseases, GO functional enrichment analysis of those genes was performed (**Figure 5A**). The differentially expressed T2DM-PRGs were mainly involved in cytokine secretion, cell junction assembly, regulation of innate immune response, positive regulation of establishment of protein localization, cell junction organization, stem cell population maintenance, calcium ion transport into the cytosol, maintenance of cell number, viral life cycle, cytosolic calcium ion transport, and other biological processes (**Figure 5B**). They were enriched in cellular components such as the cell projection membrane, COP9 signalosome, dendritic spine, neuron spine, cell leading edge, myelin sheath, sperm part, brush border membrane, sperm flagellum, and 9 + 2 motile cilium (**Figure 5C**). With regard to molecular functions, they were in high abundance for the GO terms binding to double-stranded RNA, glutamate receptors, ubiquitin protein ligases, ubiquitin-like protein ligases, and ionotropic glutamate receptors. They also showed affinity for protein phosphatase 2A and p53 (**Figure 5D**). KEGG annotation of the T2DM-PRGs revealed they were enriched in pathways related to prostate cancer, nuclear factor-kappa B (NF-κB) signaling, microRNAs in cancer, NOD-like receptor signaling, EGFR tyrosine kinase inhibitor resistance, Epstein-Barr virus infection, AGE-RAGE signaling in diabetic complications, Parkinson’s disease, thyroid hormone signaling, and autophagy. These pathways suggest the potential involvement of the T2DM-PRGs in a diverse range of diseases, including cancer, inflammation, viral infections, diabetic complications, and neurological disorders (**Figure 5E**). Specifically, the T2DM-PRGs were highly enriched in the prostate cancer (hsa05215) pathway (**Figure 5F**).

### 3.3 Gene set enrichment analysis

**Figure 6:**
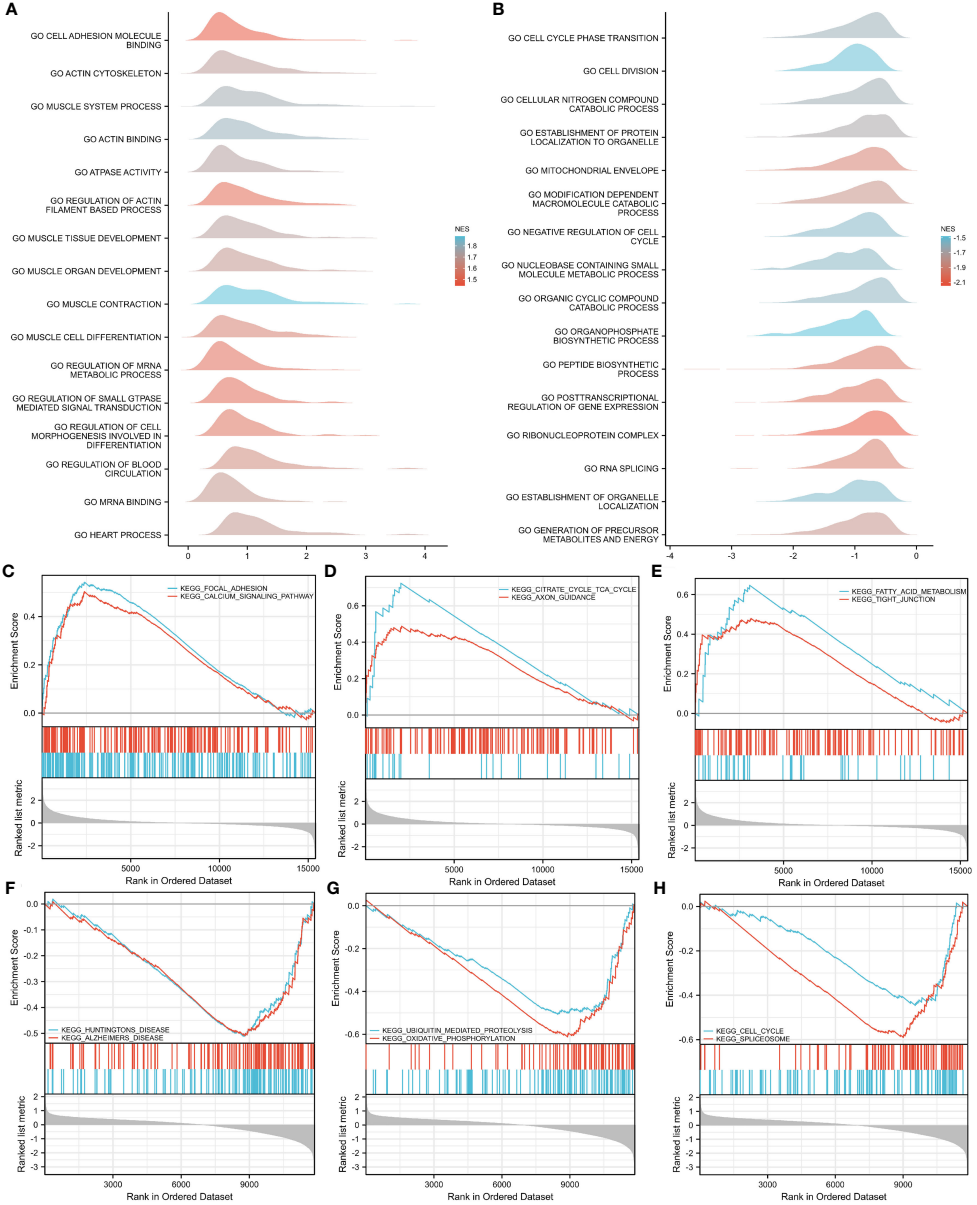
GSEA–GO and –KEGG analyses of GSE7014 and GSE25724 data. **(A, B)** GSEA–GO analysis of GSE7014 **(A)** and GSE25724 **(B)** data. In the distribution curves, the y-axis represents the gene set, the x-axis is the log fold change (FC) distribution of the core molecules in each gene set, and the shape of the peak represents the logFC of the core molecules in the gene set. The curve peak height corresponds to the position where the logFC of most molecules in this group is concentrated. If the normalized enrichment score (NES) of the corresponding gene set is negative, the peak of the gene set is generally to the left of zero; if the NES of the corresponding gene set is positive, the peak of the gene set is generally to the right of zero. **(C–E)** GSEA–KEGG analysis of GSE7014 data showing that the enriched pathways were for focal adhesion; calcium signaling; dilated cardiomyopathy; ECM–receptor interaction; hypertrophic cardiomyopathy; viral myocarditis; valine, leucine, and isoleucine degradation; fatty acid metabolism; citrate cycle; TCA cycle; and tight junctions. **(F–H)** GSEA-KEGG analysis of GSE25724 data showing that the enriched pathways are those for Huntington’s disease, Alzheimer’s disease, the cell cycle, the spliceosome, ubiquitin-mediated proteolysis, oxidative phosphorylation, and Parkinson’s disease.

To investigate the impact of gene expression levels on T2DM, GSEA was performed on the two GEO datasets to identify the correlations between gene expression and the relevant biological processes, cellular components, and molecular functions. The results showed that in the GSE7014 dataset, the genes mainly affected adhesion molecule binding, actin cytoskeletons, muscle system processes, actin binding, ATPase activity, the regulation of actin filament-based processes, muscle tissue development, muscle organ development, muscle cell differentiation, muscle contraction, and other biological functions (**Figure 6A**). The genes in the GSE25724 dataset primarily impacted critical biological functions, such as transitions between cell cycle phases, cell division, breakdown of cellular nitrogen compounds, establishment of protein localization within organelles and the mitochondrial envelope, breakdown of modification-dependent macromolecules, restraining of the cell cycle, metabolism of nucleobase-containing small molecules, breakdown of organic cyclic compounds, and biosynthesis of organophosphates (**Figure 6B**).

With regard to the biological pathways affected by gene expression in both datasets, our findings indicated that the genes in the GSE7014 dataset primarily influenced biologically pertinent ones, including those related to focal adhesion; dilated cardiomyopathy; hypertrophic cardiomyopathy; valine, leucine, and isoleucine degradation; the citrate cycle; the TCA cycle; calcium signaling; extracellular matrix (ECM)–receptor interaction; viral myocarditis; fatty acid metabolism; and tight junctions (**Figures 6C–E**). The biological pathways that were mainly controlled by genes in the GSE25724 dataset were those related to Huntington’s disease, Alzheimer’s disease, the cell cycle, the spliceosome, ubiquitin-mediated proteolysis, oxidative phosphorylation, Parkinson’s disease, the proteasome, the citrate cycle, the TCA cycle, and protein export (**Figures 6F–H**).

### 3.4 Construction of the protein–protein interaction network

**Figure 7:**
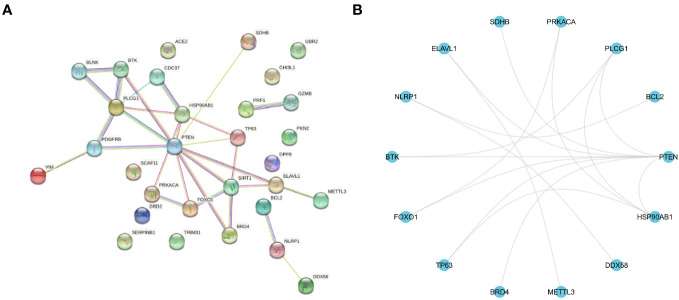
Protein–protein interaction (PPI) network. **(A)** PPI network of T2DM-associated pyroptosis-related genes (T2DM-PRGs), constructed using the STRING database. Each network node represents a protein, and the lines represent protein–protein associations. **(B)** R software-generated diagram of the PPI network of the protein interactions of T2DM-PRGs from the table provided by the STRING database. The 14 genes with the highest number of interactions are shown.

In this study, the T2DM-PRGs in the STRING database were used to construct a PPI network of the differentially expressed T2DM-PRGs, using the igraph and ggraph packages in R (**Figures 7A, B**). Cytoscape software was used for visualization of the network. There were 30 T2DM-PRG-associated DEGs and 29 PPI pairs in the generated PPI network, among which DEGs related to other T2DM-PRGs interacted with one another. The five genes with the strongest cooperative relationships were *PTEN*, *PLCG1*, *SIRT1*, *HSP90AB1*, and *TP63*.

### 3.5 Network analysis of T2DM-PRGs and related miRNAs, transcription factors, and drugs

**Figure 8:**
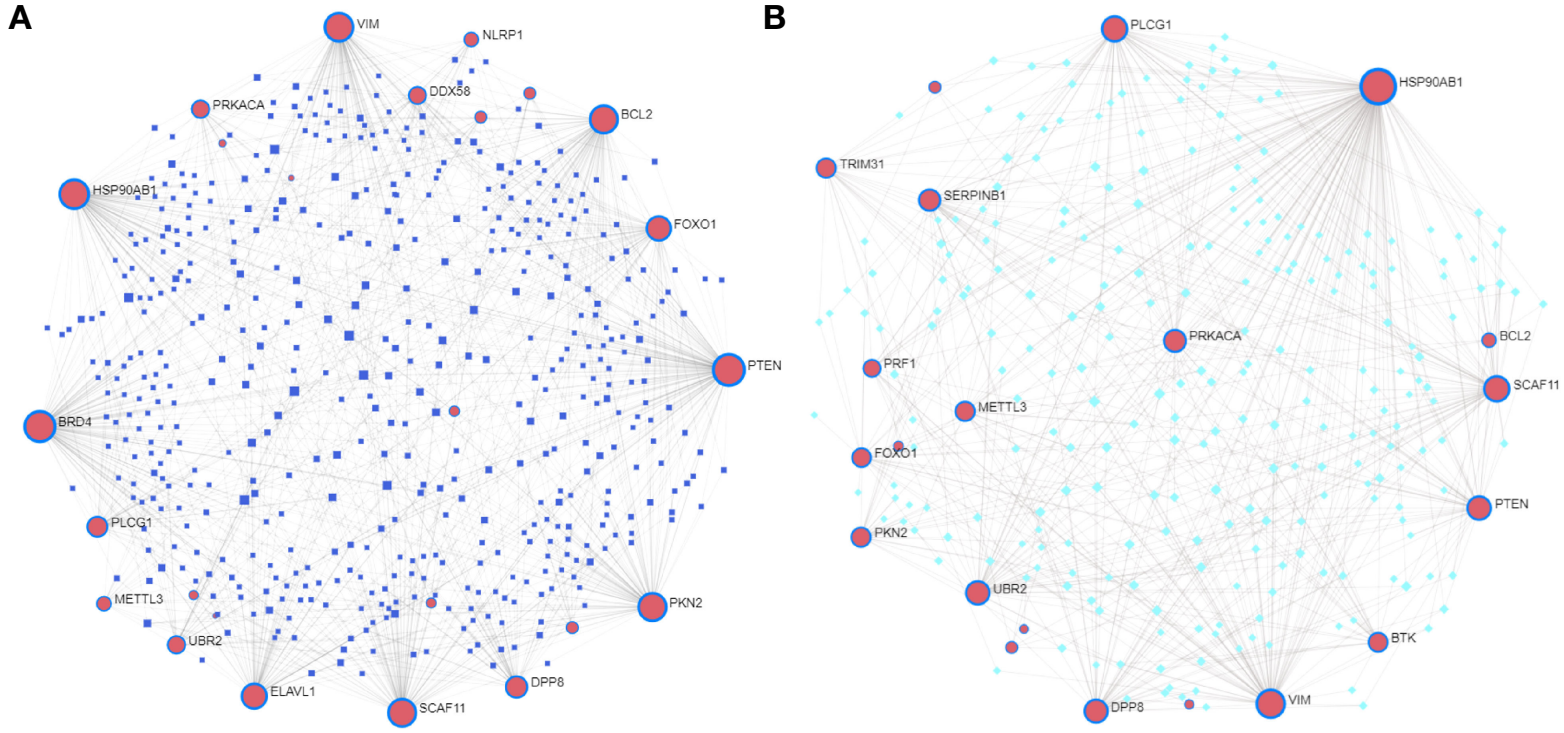
Correlations between T2DM-associated pyroptosis-related genes (T2DM-PRGs) and miRNAs and transcription factors (TFs). **(A)** T2DM-PRG–miRNA network. The blue nodes represent miRNAs, and the pink nodes represent T2DM-PRGs. **(B)** T2DM-PRG–TF network. The green nodes represent TFs and the purple nodes represent T2DM-PRGs.

We constructed a T2DM-PRG–miRNA interaction network comprising 25 genes and 512 miRNAs (**Figure 8A**). The top five differentially expressed T2DM-PRGs related to prognosis were *PTEN* (targeted by 128 miRNAs), *BRD4* (targeted by 118 miRNAs), *HSP90AB1* (targeted by 103 miRNAs), *VIM* (targeted by 96 miRNAs), and *PKN2* (targeted by 91 miRNAs).

**Figure 9:**
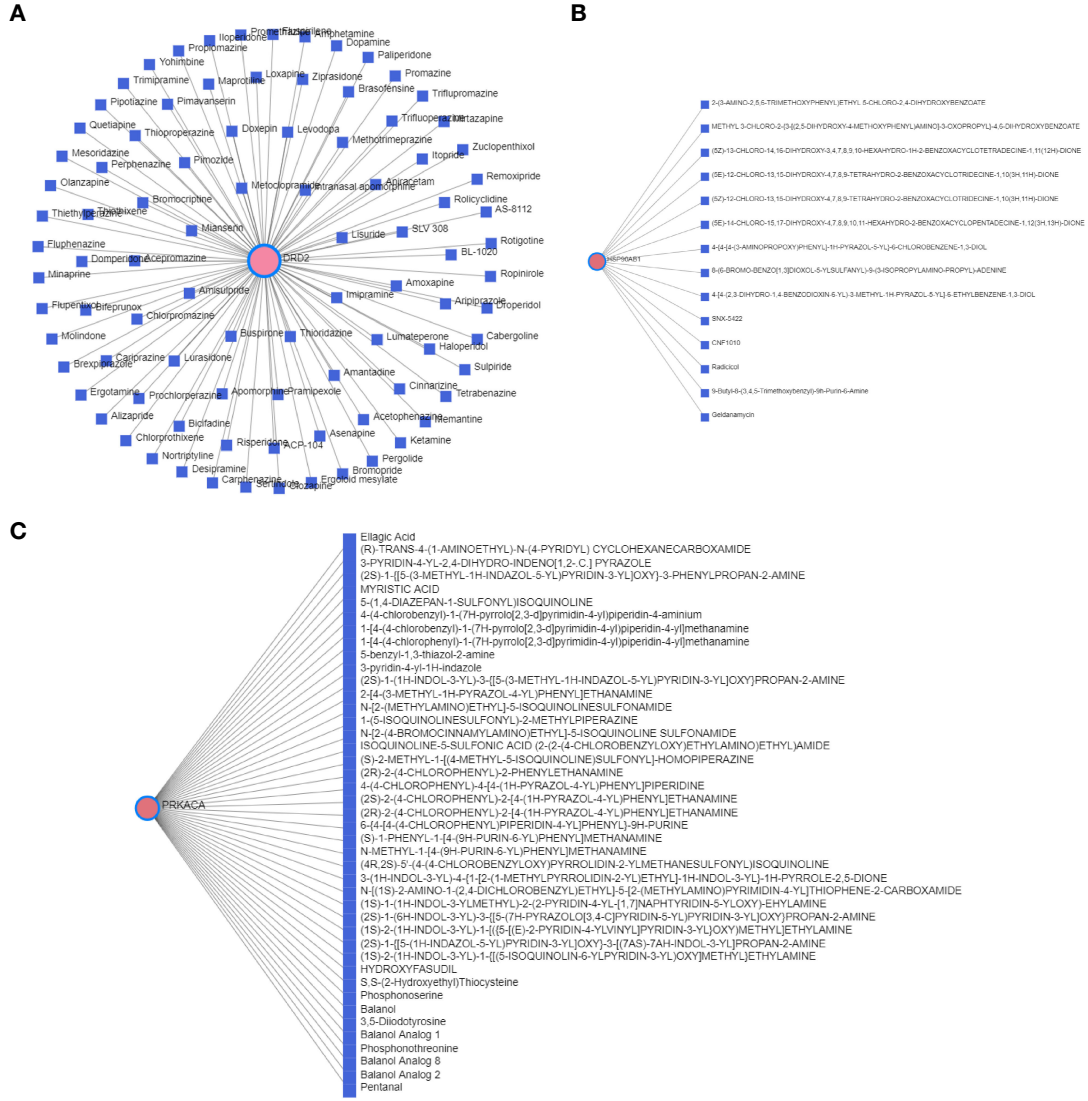
Correlation between T2DM-associated pyroptosis-related genes (T2DM-PRGs) and drugs. **(A–C)** The top three networks with the highest effect of drugs on T2DM-PRGs are displayed. The blue nodes represent drugs, and the pink nodes represent T2DM-PRGs.

The T2DM-PRG–TF interaction network comprised 21 genes and 274 TFs (**Figure 8B**). The top five differentially expressed T2DM-PRGs were *HSP90AB1* (regulated by 151 TFs), *VIM* (regulated by 75 TFs), *PLCG1* and *SCAF11* (regulated by 54 TFs each), and *PTEN* (regulated by 44 TFs). The T2DM-PRG–drug interaction network included seven networks and seven genes, of which the first three networks had 92, 44, and 15 drug effects, respectively (**Figures 9A–C**).

### 3.6 T2DM-PRG expression analysis and ROC validation

**Figure 10:**
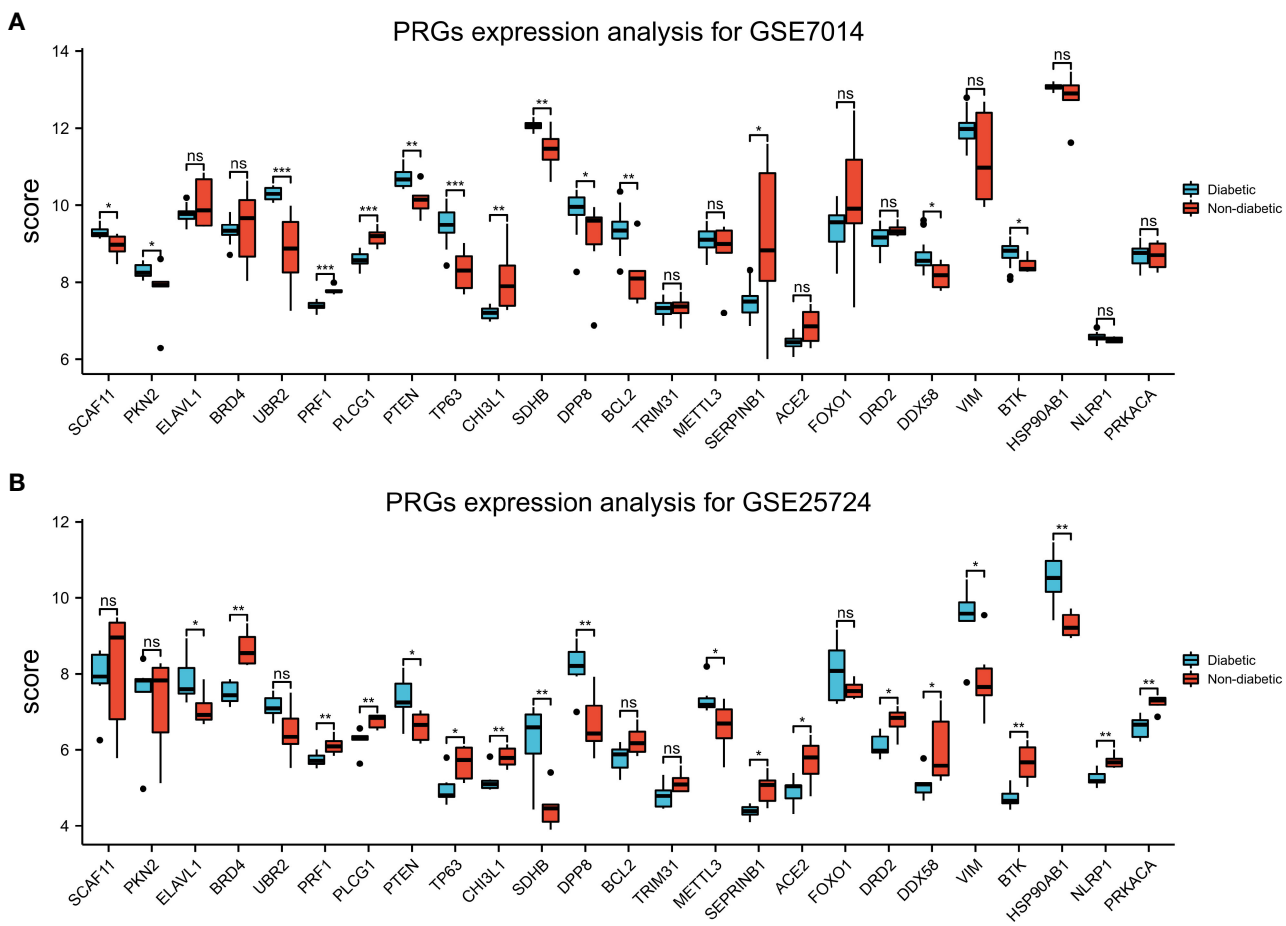
Expression of T2DM-associated pyroptosis-related genes (T2DM-PRGs). **(A)** Expression levels of the 25 T2DM-PRGs in the GSE7014 dataset. Red represents the normal group, blue represents the disease group, the abscissa represents the T2DM-PRGs, and the ordinate represents the gene expression value (p > 0.05). **(B)** Expression levels of the 25 T2DM-PRGs in the GSE25724 dataset. Red represents the normal group, blue represents the disease group, the abscissa represents the T2DM-PRGs, and the ordinate represents the gene expression value (p < 0.05; *, p<0.05; **, p<0.01; ***, p<0.001; ns, not significant).

Box plots of the expression levels of the T2DM-PRGs in the GSE7014 and GSE25724 datasets were constructed (**Figures 10A, B**). In the GSE7014 dataset, the expression levels of *BCL2*, *CHI3L1*, *DDX58*, *DP8*, *PKN2*, *PRF1*, *PLCG1*, *PTEN*, *SCAF11*, *SDHB*, *SERPINB1*, *TP63*, *UBR2*, and *BTK* were significantly different (p < 0.05). In the GSE25724 dataset, the genes with a statistically significant difference in expression level were *EACE2*, *BRD4*, *BTK*, *CHI3L1*, *DRD2*, *DDX58*, *DPP8*, *ELAVL1*, *HSP90AB1*, *METTL3*, *PRF1*, *PLCG1*, *PTEN*, *SDHB*, *SEPRINB1*, *TP63*, *VIM*, *NLRP1*, and *PRKACA* (p < 0.05).

**Figure 11:**
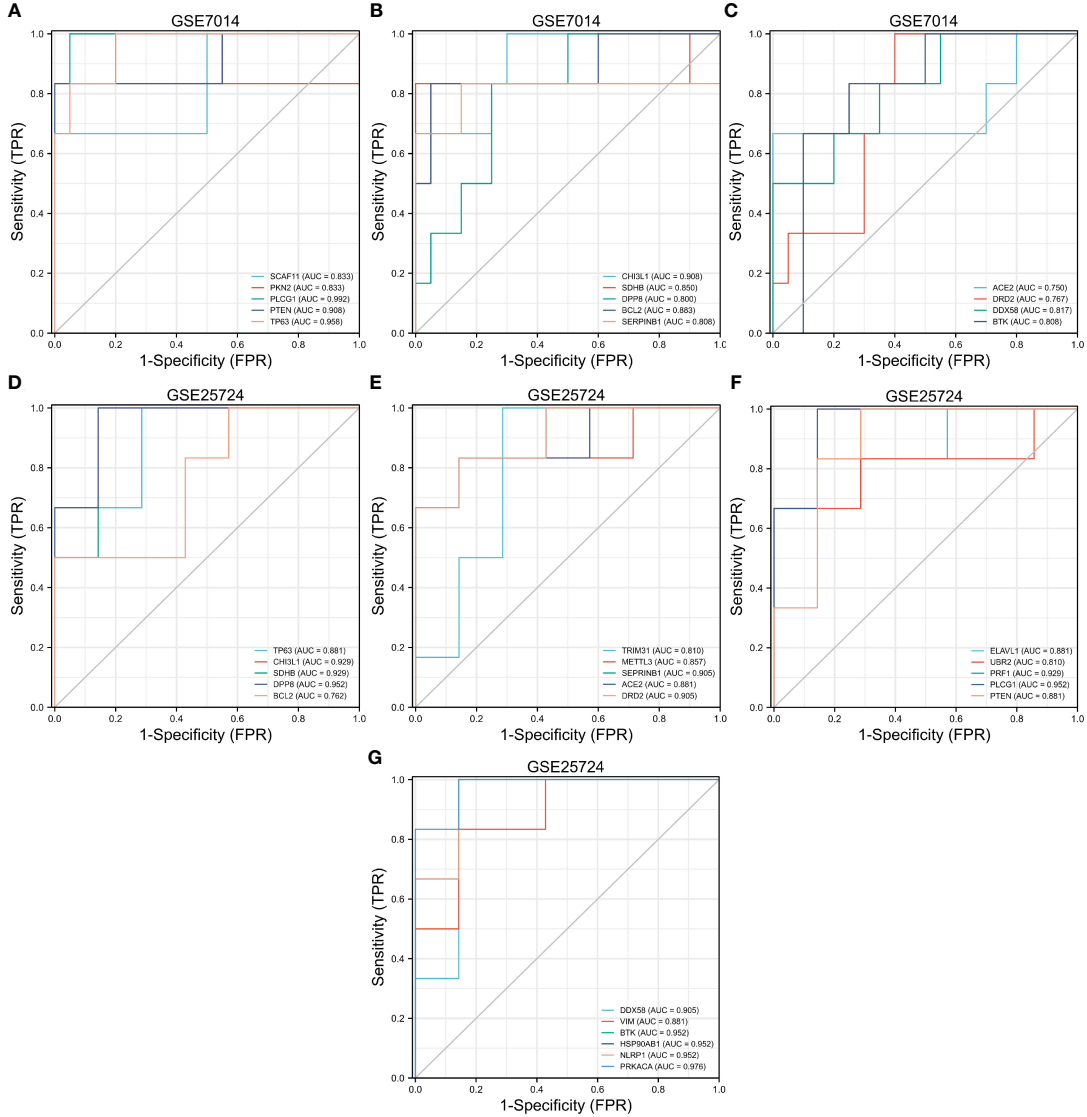
Receiver operating characteristic (ROC) curve prediction of T2DM-associated pyroptosis-related genes (T2DM-PRGs) in the GSE7014 and GSE25724 datasets. **(A–C)** ROC curves for T2DM-PRGs in the GSE7014 dataset (AUC > 0.7). **(D–G)** ROC curves for T2DM-PRGs in the GSE25724 dataset (AUC > 0.7).

In the GSE7014 dataset, the genes that exhibited diagnostic value were *ACE2*, *BCL2*, *BTKCHI3L1*, *DDX58*, *DPP8*, *DRD2*, *PKN2*, *PLCG1*, *PTEN*, *SCAF11*, *SDHB*, *SERPINB1*, and *TP63* (AUC > 0.7). In the GSE25724 dataset, the genes with diagnostic value were *TP63*, *CHI3L1*, *SDHB*, *DPP8*, *BCL2*, *TRIM31*, *METTL3*, *SEPRINB1*, *ACE2*, *DRD2*, *ELAVL1*, *UBR2*, *PRF1*, *PLCG1*, *PTEN*, *DDX58*, *VIM*, *BTK*, *HSP90AB1*, *NLRP1*, and *PRKACA* (AUC > 0.7) (**Figure 11**).

### 3.7 Immune infiltration analysis

**Figure 12:**
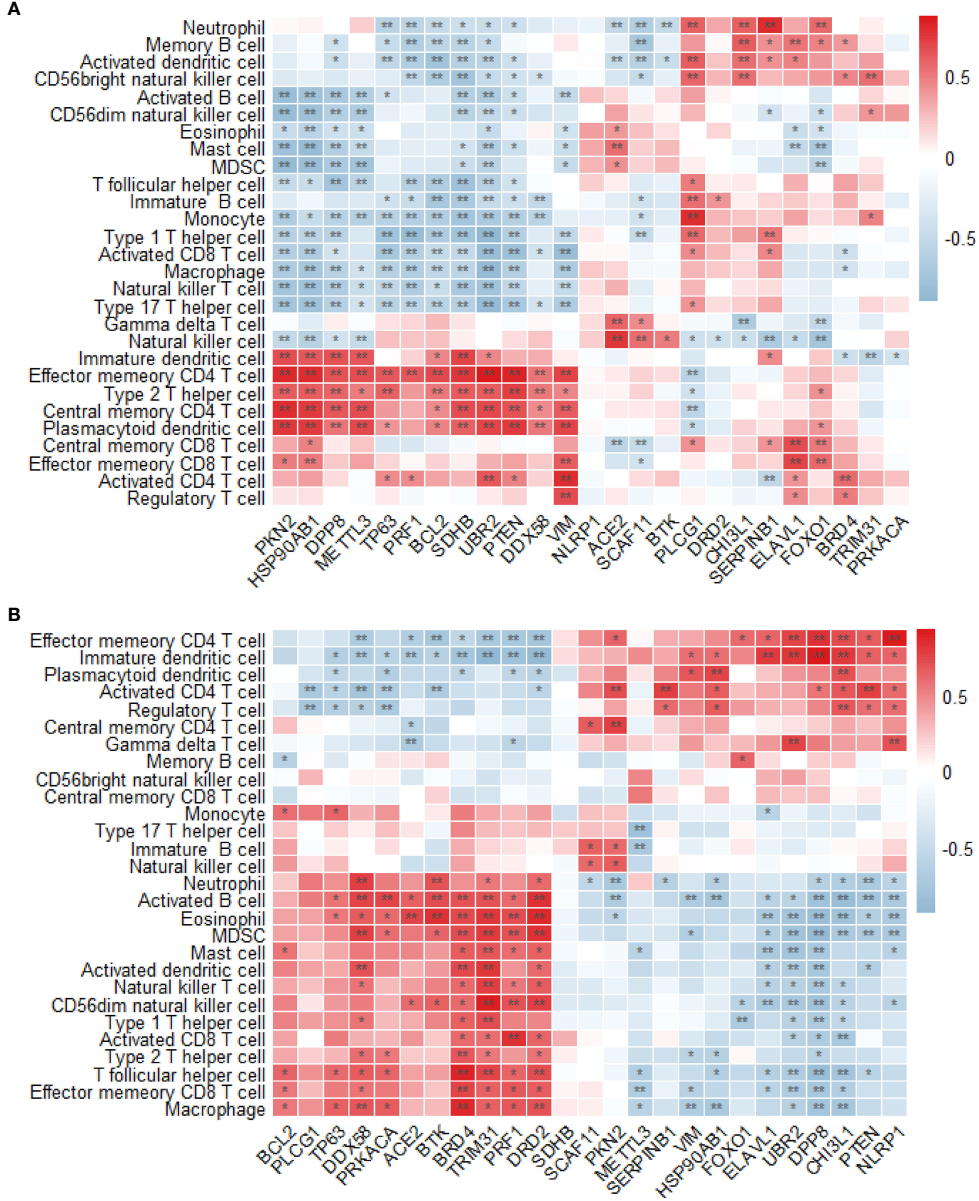
Analysis of immune infiltration. **(A)** Differences in the enrichment abundance of 28 immune cells in the GSE7014 dataset. Blue indicates a negative correlation with immune cells, red indicates a positive correlation with immune cells, the horizontal axis indicates T2DM-associated pyroptosis-related genes (T2DM-PRGs), and the vertical axis indicates immune cells. **(B)** Differences in the enrichment abundance of 28 immune cells in the GSE25724 dataset. Blue indicates a negative correlation with immune cells, red indicates a positive correlation with immune cells, the horizontal axis indicates T2DM-PRGs, and the vertical axis indicates immune cells. *, p<0.05; **, p<0.01.

The permeability of T2DM and normal tissues to immune cells was compared using the single-sample GSEA method. In the GSE7014 dataset, 28 distinct types of immune cells exhibited significant differences between T2DM and normal tissue in relation to their association with T2DM-PRGs. The genes and their associated immune cells were *ACE2* and gamma delta T cells; *BCL2* and effector memory CD4 T cells; *BRD4* and activated CD4 T cells; *CHI3L1* and activated dendritic cells, central memory CD4 T cells, and central memory CD8 T cells; *DPP8* and central memory CD4 T cells; *DDX58* and effector memory CD4 T cells and effector memory CD8 T cells; *ELAVL1* and central memory CD8 T cells; *FOXO1* and central memory CD8 T cells; *HSP90AB1* and central memory CD4 T cells, immature B cells, immature dendritic cells, mast cells, and memory B cells; *METTL3* and central memory CD4 T cells, monocytes, natural killer cells, and neutrophils; *PKN2* and central memory CD4 T cells and plasmacytoid dendritic cells; *PLCG1* and activated dendritic cells; *PRF1* and effector memory CD4 T cells; *PTEN* and central memory CD4 T cells and regulatory T cells; *SCAF11* and natural killer cells; *SERPINB1* and neutrophils; *SDHB* and central memory CD4 T cells, type 1 T helper cells, and type 2 T helper cells; *TRIM31* and CD56 bright natural killer cells; *TP63* and effector memory CD4 T cells; *VIM* and activated CD4 T cells; and *UBR2* and activated CD4 T cells and type 2 T helper cells (r > 0.5, p < 0.01) (**Figure 12A**).

In the GSE7014 dataset, we observed 28 distinct types of immune cells that exhibited significant differences between the T2DM and normal tissues in relation to their association with T2DM-PRGs. The genes and immune cells that showed significant positive correlations (r > 0.5, p < 0.01) were *PKN2* and activated CD4 T cells and central memory CD4 T cells; *ELAVL1* and immature dendritic cells; *BRD4* and activated B cells, activated dendritic cells, effector memory CD8 T cells, eosinophils, macrophages, myeloid-derived suppressor cells (MDSCs), T follicular helper cells, type 1 T helper cells, and type 2 T helper cells; *UBR2* and effector memory CD4 T cells, gamma delta T cells, and immature dendritic cells; *PRF1* and activated CD8 T cells, CD56 dim natural killer cells, eosinophils, and MDSCs; *PTEN* and activated CD4 T cells; *CHI3L1* and effector memory CD4 T cells, immature dendritic cells, plasmacytoid dendritic cells, and regulatory T cells; *DPP8* and effector memory CD4 T cells and immature dendritic cells; *TRIM31* and activated B cells, activated dendritic cells, CD56 dim natural killer cells, eosinophils, mast cells, MDSCs, natural killer T cells, T follicular helper cells, and type 1 T helper cells; *SERPINB1* and activated CD4 T cells; *ACE2* and eosinophils; *DRD2* and activated B cells, CD56 dim natural killer cells, eosinophils, macrophages, MDSCs, and T follicular helper cells; *DDX58* and activated B cells, activated dendritic cells, macrophages, MDSCs, and neutrophils; *BTK* and activated B cells, eosinophils, and neutrophils; *HSP90AB1* and plasmacytoid dendritic cells; *NLRP1* and effector memory CD4 T cells and gamma delta T cells; and *PRKACA* and activated B cells (**Figure 12B**).

## 4 Discussion

DM, a chronic disease that affects the ability of the body to control blood sugar levels and results in a range of micro- and macrovascular complications, has reached epidemic levels worldwide (50). Pyroptosis, a type of programmed cell death, is a critical process in the pathophysiology of DM and its related complications. Although the activation mechanism is unclear, our findings suggest that molecules involved in the pyroptosis and inflammasome pathways could be key in treating DM and its complications by virtue of them being targets of future drugs developed to inhibit these pathways. Necrosis is responsible for the death of most pathophysiologically important cells, whereas apoptosis maintains regular metabolism, contributing to host survival and growth as part of the body’s defense against microbial diseases (51–54). Necrosis is caused by rupture of the plasma membrane, which leads to the release of damage-associated molecular patterns and the subsequent activation of necroinflammation. Controlled necrosis includes necroptosis, pyroptosis, and ferroptosis. In this study, we focused on the pyroptosis pathway and its relationship with T2DM.

The pyroptosis signaling pathways are classified into classical and non-classical types (55). In the classical signaling pathway, inflammasomes composed of nucleotide-binding oligomerization domain, leucine rich repeat and pyrin domain containing (NLRP) 1, NLRP3, NLR family CARD domain containing 4 (NLRC4), absent in melanoma 2 (AIM2), and other proteins activate pyroptosis via membrane receptors that recognize pathogen- or damage-associated molecular patterns. The inflammasome complex controls the activation of caspase-1, thereby promoting the latter’s proteolytic cleavage of gasdermin D (GSDMD). Upon its cleavage, the activated GSDMD molecules release their N-terminal domains, the aggregation of which creates pores in the cell membrane, leading to pyroptotic cell death. The non-classical signaling pathway involves the cleavage of GSDMD by caspase-4, -5, or -11 (56). Initially, it was believed that T cell-mediated adaptive immunity was involved in the development of T1DM. However, recent studies have shown that the innate immune system, which is controlled by Toll-like receptors, is integral to the onset and pathogenesis of T1DM (57). However, despite the data obtained from mouse models demonstrating the significance of pyroptosis in innate immunity, there is a lack of research on whether pyroptosis plays a role in the etiology and progression of DM and its associated complications.

The T2DM-PRGs identified in the PPI network have similar features function and exhibit high clinical diagnostic value. The top five genes (*PTEN*, *PLCG1*, *SIRT1*, *HSP90AB1*, and *TP63*) may be considered essential target genes and can be linked to the pathogenesis of T2DM; future studies should focus on confirming this. The five most prominent differentially expressed prognostic T2DM-PRGs targeted by miRNAs were *BRD4*, *HSP90AB1*, *PTEN*, *PKN2*, and *VIM*. The top five differentially expressed T2DM-PRGs regulated by TFs were *HSP90AB1*, *VIM*, *PLCG1*, *SCAF11*, and *PTEN*. Phosphatase and tensin homolog (PTEN) activates the protein kinase B/mammalian target of rapamycin (AKT/mTOR) pathway to induce autophagy, which plays a crucial role in regulating cellular energy balance. It has been previously revealed that a decrease in circulating miRNAs indicates an increase in the transcript expression of their target genes, such as *PTEN*, which in turn can inhibit cell growth pathways, activate cell survival pathways, and promote healthy aging (58).

A previous study revealed significant differences in *HSP90AB1* mRNA expression levels between squamous cell carcinomas and healthy control tissue (59). The T2DM-PRGs–drug interaction network indicated that targeting of the dopamine receptor D_2_ (*DRD2*) gene could inhibit tumors, but the effect on T2DM is unknown. Adrenocortical adenomas that cause Cushing’s syndrome develop as a result of mutations in *PRKACA*, the gene encoding protein kinase cAMP-activated catalytic subunit alpha (PKA C-alpha) (60). A few studies have reported PRKACA-related drug targets that were not previously associated with hypoglycemic drugs.

Our KEGG analysis identified NF-κB signaling pathways as being significant in prostate cancer. We created a map of the KEGG prostate cancer pathway (entry hsa05215) and identified controlled proteins, finding drugs that could potentially affect phosphatidylinositol 3-kinase (PI3K; insulin sensitivity), glycogen synthase kinase-3 (GSK3; glycogenesis), and AKT (insulin sensitivity). Those drugs may address DM caused by insulin resistance, as shown by earlier research on PI3K, GSK3, and AKT (KEGG insulin resistance pathway; entry hsa04931) (61). In long-term DM, insulin-like growth factor 1 receptor (IGF-1R) is reduced, and elevated IGF-1 levels correlate with increased prostate cancer risk (62–64). A wide variety of human cancers have been linked to the overexpression of insulin receptors and IGF-1R resulting from transcriptional dysregulation (65–68). A previous study has demonstrated that the NF-κB, PI3K–AKT, and mTOR signaling pathways are overrepresented in DM-induced erectile dysfunction (69). Through our investigation of the T2DM-related genes using GSEA, we discovered active pathways in T2DM, including those of focal adhesion, calcium signaling, the citrate cycle, axon guidance, and fatty acid metabolism. Previous research indicates that DM and diabetic peripheral neuropathy might be highly influenced by immune, inflammatory, and focal adhesion pathways, and DM could also be greatly affected by cancer, ECM–receptor interaction, and immune-related pathways (70). In one study, circular RNAs in peripheral blood mononuclear cells of patients with diabetic retinopathy were found to be enriched in ECM–receptor interaction and focal adhesion pathways, significantly contributing to the migration of retinal vascular endothelial cells (71). This is consistent with other studies showing that mitochondrial oxidative phosphorylation is associated with peripheral nerve lesions in patients with DM.

Through functional enrichment and immune infiltration analyses, we found that the differentially expressed T2DM-PRGs were mainly enriched in biological processes and pathways related to inflammation, the immune response, and cell signal transduction. This not only emphasizes the close relationship between cell death and the immune system but also provides new clues for studying the immune mechanism of T2DM. Additionally, it has been demonstrated that pyroptosis is related to the immunological activation of the tumor microenvironment (72). In another study, a notable difference in the number of immature dendritic cells was found between diabetic and normal mice, a finding that may be due to the induction and maintenance of allogeneic tolerance by dendritic cells, which are the key regulators of the immune system. Previous research suggests that immature dendritic cells can be modified to prevent islet xenograft rejection (73). In stable dendritic cells, CD4+ memory T cell responses are suppressed (74) via the blocking of co-stimulatory molecules (75). Diabetic and healthy cells show considerable differences in effector memory CD4+ T cells, with a higher percentage observed in T2DM patients without cardiovascular disease (76). Immune infiltration analysis further supported this finding, showing a notable difference in the infiltration of various immune cells in T2DM tissue, which was related to T2DM-PRGs. For example, the infiltration of natural killer cells into T2DM tissue was significantly increased. This may be related to immunoregulation and inflammatory reactions associated with T2DM. The numbers of central memory CD4+ T cells and effector memory CD4+ T cells were substantially increased in diabetic tissue, suggesting that T cells play an important role in the immune response against T2DM. Activated dendritic cells and plasmacytoid dendritic cells were also significantly increased in the diabetic tissue, which may be related to the activation of immune system and inflammation.

Furthermore, *NLRP1* and *PRKACA* showed substantial differences in their expression levels and diagnostic value. Notably, *PLCG1* and *DPP8* showed excellent discriminative abilities in the prediction of diabetes. The precise relationship between 1-phosphatidylinositol 4,5-bisphosphate phosphodiesterase gamma-1 (PLCG1) and dipeptidyl peptidase 8 (DPP8) and the underlying mechanisms of action in the development of T2DM have yet to be explored. However, according to the results of our study, low *PLCG1* and high *DPP8* expression levels were associated with worse survival outcomes, which could be due to increased pyroptotic activity. PLCG1 is a member of the phosphatidylinositol-specific phospholipase C family, a group of membrane-associated enzymes that cleave phosphatidylinositol 4,5-bisphosphate into diacylglycerol and inositol 1,4,5-trisphosphate and can cause cell death and inflammatory responses by releasing intracellular calcium reserves (77). PLCG1 is involved in signal transduction pathways triggered by receptor tyrosine kinases, regulating GSDMD activity and promoting apoptosis. Low PLCG1 levels disrupt GSDMD-induced pyroptosis (78), whereas high levels have been observed in diabetic rats and high glucose-treated retinal endothelial cells (79). However, one study suggested that DPP8, a cytosolic protease linked to cancer biology and N-terminal dipeptidyl peptidases, is a potential therapeutic target for T2DM, albeit information on its 3D structure or binding mechanisms was lacking (80). Although the physiological roles of these genes are not fully understood, we speculate that they may be involved in the intricate cellular stress process in T2DM. Considering the aforementioned factors, it is possible that these genes have a significant impact on DM and can serve as diagnostic biomarkers; further analyses are required to assess their effects.

This study had some limitations. First, microarray samples collected at various stages of T2DM are required to fully understand the molecular mechanisms underlying the occurrence and development of the disease. Additionally, the biomarkers and metabolic pathways identified using bioinformatic methods need to be confirmed with further experimental studies. Second, we used the limma and clusterProfiler packages and GSEA for differential expression analysis, functional enrichment analysis, and GSEA, respectively. However, these methods have certain limitations. For example, differential expression analysis can only provide information regarding the differences in expression level of the genes and cannot infer their specific functions and mechanisms. By contrast, functional enrichment analysis can only provide the biological function associations of the gene sets, and there may be omissions and misinterpretations of the available information. Third, the sample sources were limited to human skeletal muscle biopsies and pancreatic islet tissue samples, which may not cover all the tissues and cell types associated with T2DM. Subsequent investigations are required to determine whether these findings can be applied to other tissues. Fourth, we constructed PPI networks and gene–miRNA, gene–TF, and gene–drug interaction networks to explore the regulatory and molecular associations of T2DM-PRGs. However, the results of these network analyses were based only on known interactions, and there may be unknown regulations and associations. Additionally, network analyses may have errors and biases. Finally, the relationship between pyroptosis and genetic characteristics requires further exploration. Therefore, further independent studies are required to confirm and strengthen the translation of our study findings to therapeutic applications.

In summary, our comprehensive bioinformatics analysis of T2DM datasets (GSE7014 and GSE25724) identified 25 differentially expressed T2DM-PRGs and revealed a connection between pyroptosis-related pathways and T2DM. Our findings provide potential therapeutic targets and diagnostic biomarkers that will help improve the management and treatment of T2DM patients. Thus, our study has positive clinical significance and scientific value in the field of T2DM. Additionally, our findings strengthen our understanding of the relationship between pyroptosis and T2DM, providing a new direction for future research.

## Data availability statement

The datasets presented in this study can be found in online repositories. The names of the repository/repositories and accession number(s) can be found in the article/**Supplementary Items**.

## Author contributions

WW performed the literature search and data analysis. YW conceived and designed the project. WW wrote the paper. YW reviewed and amended the manuscript. All authors contributed to the article and approved the submitted version.
