# Supplementary material for: Integrative bioinformatics analysis of biomarkers and pathways for exploring the mechanisms and molecular targets associated with pyroptosis in type 2 diabetes mellitus
Source: Front Endocrinol (Lausanne). 2023 Nov 15;14:1207142. doi: 10.3389/fendo.2023.1207142 (PMC10684677; doi:10.3389/fendo.2023.1207142)
Supplement: Supplementary file 1 [file Table_1.pdf]

Supplementary Material

Integrative analyzes of biomarkers and pathways for exploring the mechanisms and targets associated with pyroptosis in type 2 diabetesFirst Author*, Co-Author, Co-Author

*** Correspondence:** Yao Wang wang_yao100@163.com

1 Supplementary Table 1

Supplementary Table 1. A total of 356 pyroptosis-related genes (abbreviated as

Pyroptosis
AIM2
CASP1
CASP3
CASP4
CASP5
CASP6
CASP8
CASP9
ELANE
GPX4
GSDMA
GSDMB
GSDMC
GSDMD
GSDME
IL18
IL1B
IL6
NLRC4
NLRP1
NLRP2
NLRP3
NLRP6
NLRP7
NOD1
NOD2
PJVK
PLCG1
PRKACA
PYCARD
SCAF11
TIRAP
TNF
GZMB
GZMA
DHX9
NLRP9
NAIP
HMGB1
KCNQ1OT1
FOXO3
MALAT1
APIP
TXNIP
GBP1
MIR214
NEK7
GJA1
P2RX7

MIR30C1
MIR22
TP53
MALT1
AGER
TET2
MIR125A
MIR155
EEF2K
PD-L1
FGF21
KLF3-AS1
CEBPB
TFAM
MEG3
MIR21
MIR135B
MIR485
STK4
PRDM1
PRF1
MST1
ELAVL1
TREM2
CDKN2B-AS1
MIR9-1
MIR9-2
MIR9-3
MIR497
HDAC6
SQSTM1
IRF3
STING1
HNP1
ZBP1
PECAM1
DDX3X
PRTN3
SERPINB1
MRE11
PARP1
CTSG
GBP5
MKI67
IL36G
IL36B
CPTP
BNIP3
ANO6
MIR103A2
MIR103A1

FADD
MEFV
APOL1
VIM
CAPN1
JUN
XIST
MIR139
ALK
SIRT1
BIRC3
BIRC2
UBE2D2
LY96
RIPK3
GLMN
IRGM
NLRP13
TUBB6
NOS2
NOS1
PYDC2
IFI16
AKT1
EGFR
TP63
ATF6
IRF1
IRF2
POP1
ORMDL3
MDM2
BTK
NFKB1
STAT3
BCL2
TLR2
ANXA2
IL1RN
BECN1
CD14
GSTO1
IL13
CHI3L1
PANX1
LRPPRC
CXCL8
IL13RA2
IL32
BST2
GPER1

LYST
CLEC5A
GAS5
MIR223
MIR15A
MIR20B
NR1H2
CAMP
BAK1
BAX
CHMP2A
CHMP2B
CHMP3
CHMP4A
CHMP4B
CHMP4C
CHMP6
CHMP7
CYCS
IL1A
DFNA5
CASP11
NLRP12
MYD88
RIG-I
DDX58
STING
TAK1
MAP3K7
RIPK1
P2X7
DPP8
DPP9
SCGB3A2
SDC1
TOM20
PELP1
CARD8
NF-kB
P53
APAF1
LXRbeta
JNK
MAPK8
PKR
CIAP1
CIAP2
TLR4
EDC2
BHLHE41
CD73

NT5E
PI3K
AKT
FOXO1
P38-MAPK
MAPK14
VDR
SYK
BDNF
KLF2
HK1
RP105/CD180
NLRX1
ASK
TRAF6
BAK
NFE2L2
MIR556
UBR2
PCSK9
BRD4
IKBKE
PKM
CRTAC1
CTSV
UTS2
MLKL
APOE
SDHB
CD274
DLX6-AS1
MIR23A
BSG
PGF
SLC16A4
CHRFAM7A
MIR124-1
MIR195
GSK3B
PTGS2
TRIM24
MPEG1
MIR204
HOTTIP
EPHA2
ABL1
CDK9
TREM1
TSLP
ZDHHC1
PTEN

DRD2
ADORA1
ADORA2B
ADORA2A
ADORA3
METTL3
TRIM31
METTL14
MIR25
FNDC4
FNDC5
TRIM21
PRKN
DUOX1
FOXP3
MIR107
VCAM1
SESN2
BHLHE40
TFAP2A
E2F4
ADAMTS9-AS2
NINJ1
TLR8
PKN2
DPEP1
CHMP1A
ACE2
LINC00958
MIR4306
YWHAE
HSP90AA1
NEDD4
HSP90AB1
IRAK3
MELK
YWHAZ
STXBP2
UBE2D3
TLR9
IFIH1
HUWE1
TNFSF13B
RAB5A
ASIC1
BRCC3
ATG7
ERP44
CDC37
TRPM2
PDCD6IP

VPS4B
ATG3
STXBP3
VPS28
NCR1
IL27
SEC22B
SIGLEC14
CGAS
F7AMM8
G3QNY2
A0A2I2YG62
LOC100068406
G1KWU3
F7GCS6
F6T749
F6S7R9
LOC102165484
G3QQR5
G3QV30
CASPA
CASPB
GSDMEA
GSDMEB
NAIP2
RGD1359449
NLRP1A
NAIP6
NLRP1B
NAIP7
URR2
GSDMA2
GSDMC4
NLRP9B
GSDMC2
CASP1-A
GSDMA3
GSDMC3
NAIP1
NAIP5
CASP1-B
IPAH7.8
LOC100068503
A0A287A923
W5MJZ2
W5MJV9
W5LZG1
W5M4Z5
A0A3Q1N5B5
K7E5T3
F7GCD9

J9NVR2

PRGs)
